# Ultrastructural differences in pretangles between Alzheimer disease and corticobasal degeneration revealed by comparative light and electron microscopy

**DOI:** 10.1186/s40478-014-0161-3

**Published:** 2014-12-11

**Authors:** Shinsui Tatsumi, Toshiki Uchihara, Ikuko Aiba, Yasushi Iwasaki, Maya Mimuro, Ryosuke Takahashi, Mari Yoshida

**Affiliations:** Department of Neuropathology, Institute for Medical Science of Aging, Aichi Medical University, Nagakute, Aichi Japan; Laboratory of Structural Neuropathology, Tokyo Metropolitan Institute of Medical Science, 2-1-6 Kamikitazawa, Setagaya, Tokyo 156-8506 Japan; Department of Neurology, Kyoto University, Kyoto, Japan; Department of Neurology, Higashi Nagoya National Hospital, Nagoya, Aichi Japan

## Abstract

Pretangles are defined under the light microscope as diffuse and granular tau immunoreactivity in neurons in tissue from patients with Alzheimer disease (AD) or corticobasal degeneration (CBD) and are considered to be a premature stage before neurofibrillary tangle formation. However, the ultrastructure of pretangles remains to be described. To clarify the similarities and differences between pretangles from patients with AD and CBD (AD-pretangles and CBD-pretangles, respectively), we examined cortical pretangles in tissue from patients with each of diseases. For direct light and electron microscopic (LM/EM) correlation of the pretangles, we used quantum dot nanocrystals (QDs) with dual fluorescent and electron-dense properties. We first identified tau-labeled pretangles on fluorescence LM and subsequently examined the same neurons on EM. Energy dispersive X-ray spectrometry (EDX) color mapping identified selenium (Se) and cadmium (Cd) as elementary components of QDs and highlighted each QD particle clearly against gray-scale EM images. With these methods, we were successful for the first time in demonstrating accurately that LM-defined pretangles are tau-positive straight filaments sparsely distributed throughout neuronal cytoplasm and neurites in both AD and CBD at the EM level. Notably, AD-pretangles showed a strong tendency to form fibrillary tangles even at an early stage, whereas pretangles or Pick-like inclusions in tissue from patients with CBD did not even at an advanced stage. In conclusion, AD-pretangles and CBD-pretangles showed essential differences at the EM level.

**Electronic supplementary material:**

The online version of this article (doi:10.1186/s40478-014-0161-3) contains supplementary material, which is available to authorized users.

## Introduction

Changes that occur in relevant molecules before they become organized into disease-specific inclusions in human brains are attracting increasing attention [1]. The pretangle is an example of such an early change; it was originally defined under light microscopy (LM) as diffuse and granular tau immunoreactivity in the cytoplasm and neurites of otherwise intact neurons in brains from patients with Alzheimer disease (AD) [2-4]. Mature neurofibrillary tangles (NFTs), which are hallmarks of AD, are readily identified as bundles of paired helical filaments (PHFs) under electron microscopy (EM) [5]. However, it is difficult to identify pretangles under EM because their faint tau immunoreactivity suggests that their ultrastructure is less distinct. Although putative ultrastructures of pretangles in AD have been reported, it is not yet clarified whether they really represent neurons containing diffuse and granular tau immmunoreactivity as defined under LM [2]. Similar granular tau immunoreactivity has also been observed in corticobasal degeneration (CBD), a four-repeat tauopathy that causes degeneration of the cerebral cortex, basal ganglia, and substantia nigra. Because they appear similar to AD-pretangles under LM, this granular tau immunoreactivity is also known as pretangles. Pretangles are considered one of the most important neuronal cytopathologies in CBD [6,7] but are also found in argyrophilic grain disease or progressive supranuclear palsy [8].

The aim of this study was to visualize the ultrastructures of LM-defined AD- and CBD-pretangles and thereby to identify possible differences between them. For this purpose, it is necessary to directly compare LM and EM images of the same pretangle, an approach that is now named “correlative light and electron microscopy (CLEM)”. Although CLEM protocols have been developing [9-17], they usually allow LM/EM comparisons for only small fields (the size of the EM preparation). This limitation makes it practically impossible to capture pretangles for immunoEM study because pretangles are not sufficiently frequent to be included by chance in such tiny preparations. Therefore, it is necessary to excise tissue containing a pretangle from the LM sample before it can be prepared for EM.

Quantum dots (QDs) are fluorescent, electron-dense semiconductor nanocrystals of uniform size with a core of cadmium selenide [18]. On EM examination, QDs also display a characteristic peripheral halo [19]. These dual optical properties allow QDs to be identified under both LM and EM and therefore permits labeled LM structures to be compared directly with their ultrastructures [16]. Using QDs, we recently established three dimension (3D) - oriented immunoelectron microscopy [19,20]. In this method, a thick floating section from the formalin-fixed human brain is incubated with the primary and QD-conjugated secondary antibodies. After a target neuron is examined with fluorescent LM (confocal microscopy), landmarks are punched out around the neuron using laser microdissection. Then, this floating section is processed for EM preparation. The advantage of this stepwise LM-EM approach is that the neuron of interest can be observed closely on confocal microscopy prior to the EM examination, and its EM findings can be supplemented with confocal images because the same reporter (i.e., QDs) can been seen under both LM and EM immunostaining.

Although QDs provide a powerful bridge between LM and EM, their electron density is lower and their contour is less distinct than those of gold particles, leading to doubts about QD use as an immunolabeling material for EM. We previously overcame this problem using energy dispersive X-ray (EDX) spectrometry, which demonstrated parallel peaks corresponding to selenium (Se) and cadmium (Cd) on the pixels for definitive confirmation of QDs on EM preparations [19]. Because it is possible to obtain EDX spectrum for each pixel, we extended this pixel-based EDX analysis to plot the entire EM field pixel by pixel in this study. Operational display of pixels containing Cd peak or those containing Se peak highlighted QD particles based on their elemental composition with different colors. When it was overlaid onto the conventional gray-scale EM image, this EDX color mapping clearly distinguished QDs from background structures such as ribosomes.

With these methods, it is possible to examine the ultrastructural details of AD- and CBD-pretangles and to elucidate their similarities and differences at the EM level [2-4,6]. Using this LM/EM correlation with novel mapping method, we obtained an EM image of the early stage of neuronal tau deposition in AD-pretangles and found essential differences between AD- and CBD-pretangles at the EM level. This is the first successful demonstration of their ultrastructural differences.

## Materials and methods

### Alzheimer disease and corticobasal degeneration cases

To investigate the ultrastructure of pretangles, we compared them in different diseases with different severities (Table 1). We used samples from one case of AD, a case of normal aging, and two typical cases of CBD. The diagnoses of AD and CBD were based on the current diagnostic criteria [6,21]. Identification of pretangles was based on LM findings as “cytoplasmic diffuse and granular tau immunoreactivity without apparent fibrillary structures”. In the normal aging samples, pretangles and Alzheimer-type NFTs were localized to the hippocampus and the parahippocampal cortex. In the CBD samples, we examined pretangles and densely packed round inclusions (Pick-like inclusions) [6] in the frontal lobe.

**Table 1:** **Demographic features of cases with AD, normal aging, and CBD**

	**Pathological diagnosis**	**Age of death (y)/sex**	**Brain weight (g)**	**Duration (y)**	**Braak NFT stage**	**Clinical symptoms**	**Type of tau-positive inclusions investigated**
Case 1	Normal aging	73/F	1,260	na	I	No history of dementia or motor symptoms	Pretangles, NFTs
Case 2	AD	58/F	850	14	VI	Severe dementia, disorientation	Pretangles, NFTs
Case 3	CBD	60/M	1,145	7	I	Supranuclear gaze palsy, frequent fall, parkinsonism, frontal signs	Pretangles, Pick-like inclusions*
Case 4	CBD	70/F	770	11	III	Frontotemporal dementia, parkinsonism	Pretangles, Pick-like inclusions, ballooned neurons

*Pick-like inclusions denoting densely packed round inclusions usually observed in the small-sized cortical neurons in corticobasal degeneration; AD, Alzheimer disease; CBD, corticobasal degeneration; NFT, neurofibrillary tangle.

### Pre-embedding tau/QD labeling for LM/EM observation

Formalin-fixed brains were rinsed in phosphate-buffered saline (PBS) and cryoprotected in 20% sucrose/PBS overnight. The tissue was frozen in optimal cutting temperature (OCT) compound and cut into 25-μm-thick floating sections on a freezing microtome. The sections were immersed in 1% bovine serum albumin/PBS for 30 min and then incubated in anti-PHF tau antibody (AT8, mouse, monoclonal, 1:700; Thermo Fisher Scientific, Tokyo, Japan) for 24 hours at room temperature (RT). After washing in PBS for 30 min, sections were incubated in an anti-mouse secondary antibody conjugated to Q-dot 655 (QD 655) (goat, 1:100 to 1:800, diluted in PBS; Invitrogen, Carlsbad, CA) for 8 hours at RT. A QD 655 dilution at 1:400 (Additional file 1: Figure S1) for a CBD pretangle provided appropriate immunoEM labeling on tau-positive filaments, whereas its fluorescent signal was not intense enough to delineate subcellular details under confocal microscopy (Additional file 1: Figure S1). Therefore, the QD-labeled sections were subsequently incubated in an anti-mouse secondary antibody conjugated to Alexa 488 (goat, 1:200; Molecular Probe) for 3 hours at RT to allow more detailed LM observation. After incubation, sections were rinsed in PBS and then mounted in fluorescence-mounting medium (S3023; Dako, Glostrup, Denmark).

### Confocal LM observation and EM preparation

**Figure 1:**
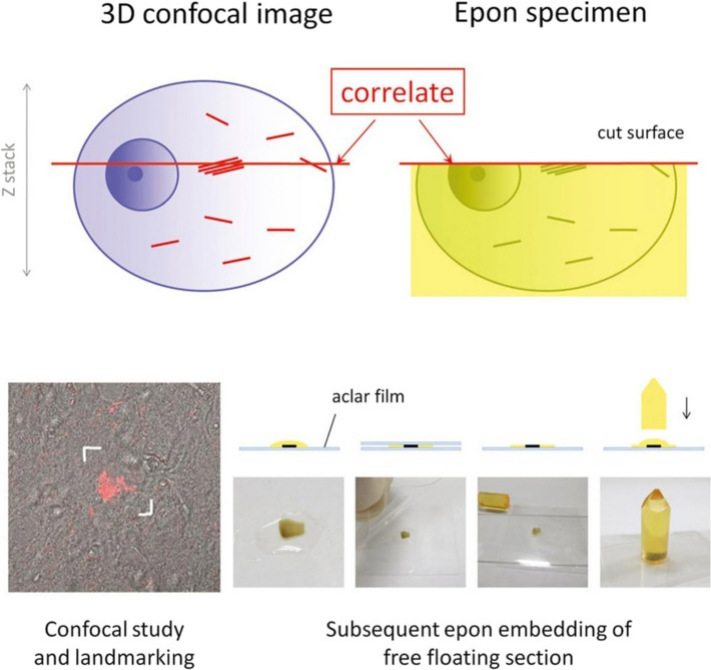
**Outline of correlation of confocal and EM images.** Three-dimensional (3D) reconstructed confocal data of the pretangles were obtained from a free-floating section. After landmarks were punched out around the target neuron using laser microdissection, sections were fixed and embedded in epon: the section was gently pressed between aclar films, hardened, then stuck to the columnar epon prepared in advance. The epon block was trimmed by the guidance of landmarks around the neuron, and ultrathin section of the block were examined with electron microscopy (EM). After obtaining the most appropriate EM images, their exact light microscopic counterparts were retrieved from the corresponding fluoresent 3D data set for direct comparison.

Three-dimensional reconstruction images of the pretangles were obtained under fluorescence confocal microscopy (LSM 710; Carl Zeiss, Oberkochen, Germany) using a 63 × −oil immersion objective lens. Alexa 488 was excited using an argon laser (488 nm), and the detection bandwidth was set at 493 to 601 nm (expected peak at 520 nm). QD 655 was excited with a diode laser (405 nm), and the detection bandwidth was set at 605 to 690 nm (expected peak at 655 nm), which gave essentially the same image as obtained with Alexa 488 [19]. A Z-series scan (800 × 800 pixels, interval 0.40 μm, approximately 10-μm thick in total) was performed for 3D reconstruction. After obtaining 3D data sets with the confocal microscope, landmarks were punched out around the target neurons using the UV laser Micro dissection system PALM (P.A.L.M. Microlaser Technologies, Bernried, Germany) (Figure 1). The sections were then detached from the glass slide, fixed in 2% glutaraldehyde for 10 min, and postfixed in 1% osmium tetroxide for 30 min. Next, the sections were embedded in epon as follows: they were gently pressed between aclar films (Nissin EM catalog #4513, Tokyo, Japan) so that flat preparation on epon was possible and then hardened with heat (60°C). An aclar film on one side was detached from the hardened epon-embedded section. Then, the section was stuck to columnar epon that had been prepared in advance (Figure 1). The target-oriented trimming of the epon block was facilitated by the guidance of punched out landmarks around the target already identified and 3D-reconstructed on confocal microscopy. Ultrathin sections of the trimmed blocks were stained with uranyl acetate/lead citrate and examined with a JEM-1400 electron microscope (JEOL, Tokyo, Japan). After obtaining the most appropriate EM images, their exact LM counterpart was retrieved from the corresponding fluorescent 3D data set for direct comparison (Figure 1).

### Energy dispersive X-ray (EDX) spectrometry and elemental mapping of QDs

The EM sections were also observed under a Hitachi HD-2700 scanning transmission electron microscope (STEM, Hitachi High Technologies Corporation, Tokyo, Japan). This STEM is equipped with a cold-field emission gun and detectors that consist of bright-field, high-angle annular dark-field (HAADF) and secondary electron (SE) detectors, which distinguish different elements (Cd and Se in this experiment) based on their energy spectra on a pixel basis. This approach identifies the presence of Cd and Se in each STEM pixel. This pixel-based identification of Cd and Se is then extended to map the entire EM field to delineate the QD particles in relation to underlying ultrastructures. The STEM was operated at 200 kV and an EDX spot analysis was performed with an incident beam size of 0.2 nm and a current of 0.4 nA. The acquisition time for each pixel was 200 μsec. In the EDX mapping, the EDX analysis was performed in a 0.4 μm × 0.5 μm field, and the total acquisition time was 90 min. Pixels containing Se or Cd peaks were displayed on the EM field independently in different color channels.

## Results

### EDX analysis and EDX mapping of QDs

**Figure 2:**
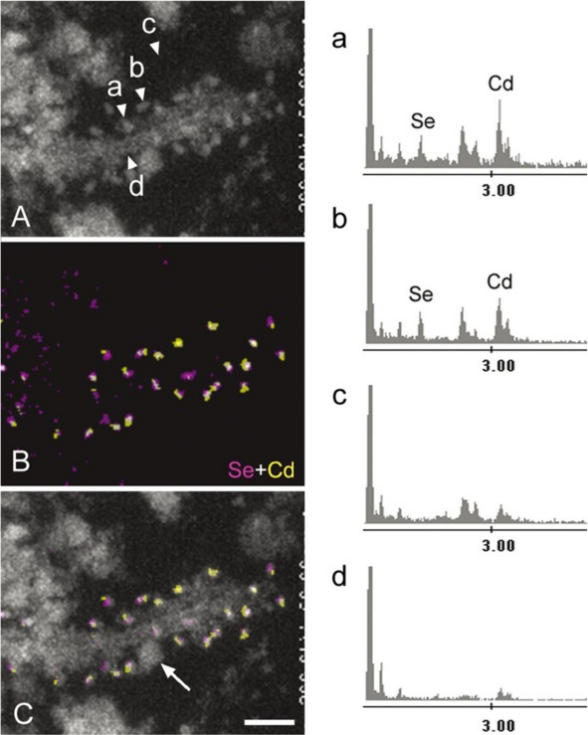
**EDX mapping of QDs around tau-positive straight filaments in a case of CBD.** EDX spot analysis of the section highlighted energy peaks corresponding to Se and Cd on QDs (Arrowheads **a** and **b** in panel **A** correspond to energy spectra **a** and **b**), but not on the background (arrowhead **c**) and the filament itself (arrowhead **d**). This pixel-based identification of Cd and Se is then extended to map the entire EM field to delineate the QD particles in relation to underlying ultrastructures. This approach produced Se (pink)- and Cd (yellow)-specific signals derived from QDs **(B)** as a map independent of underlying ultrastructures. The QDs were easily distinguished from a ribosome (arrow) (overlay, **C)**. Scale bar = 50 nm.

The shape of electron-dense QD 655, dribbled on the formvar membrane, was spherical to oblong on the STEM image (Additional file 2: Figure S2A). Pixel-based EDX elementary mapping highlighted the distribution of Se and Cd (Additional file 2: Figure S2B and D, respectively), which exactly corresponded to the ultrastructural shape of QDs (Additional file 2: Figure S2C). In a CBD-pretangle (case 3) examined using a QD 655-conjugated secondary antibody, tau filaments were labeled with numerous QDs of similar morphology (Figure 2A). The EDX spot analysis identified these QDs on the basis of energy peaks corresponding to Se and Cd. Elemental mapping with this EDX analysis further showed the distribution of QDs based on the presence of Se and Cd (Figure 2B). As mentioned, when the original STEM image was overlaid onto this EDX mapping, the regional distributions of these analytical QD signals were identical to the ultrastructural shapes of QDs (Figure 2C). With this technique, the QDs could be readily differentiated from the grayscale background, even if the tau-filaments were intermingled with (similarly round) ribosomes (Figure 2C, arrow).

### LM findings and corresponding ultrastructures of pretangles in AD and aging

**Figure 3:**
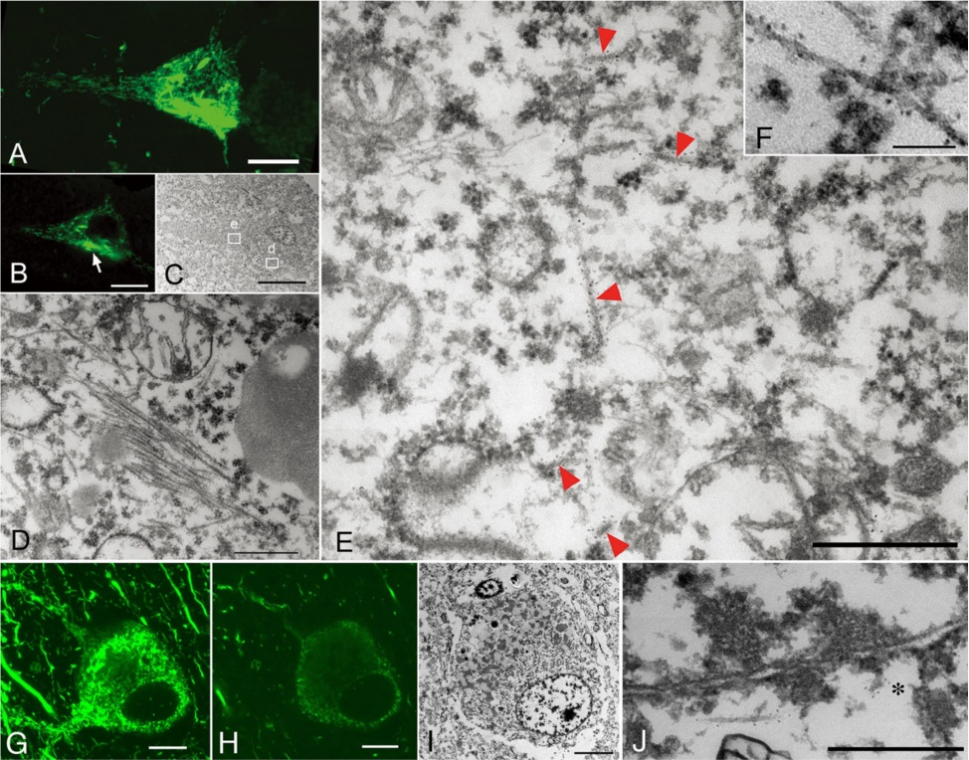
**Correlation of immunolabeled LM and EM images of Alzheimer disease (AD)-type pretangles.** A mixture of granular tau immunoreactivity and tangle-like aggregates in a pretangle neuron from a normal aging brain (case 1) (**A**, stacked 3D image). This neuron was labeled with anti-tau antibody(AT8) visualized with QD 655, also labeled with Alexa 488 for clearer confocal images. When an EM section **(C)** and the corresponding LM counterpart on the same plane **(B)** were compared, the tangle-like aggregate (**B**, arrow; **C**, rectangle d) was found to be a small bundle of straight and paired helical filaments tightly arranged in parallel **(D)**. Granular immunoreactivity on LM (**C**, rectangle e) corresponded to 15-nm straight filaments widely and randomly distributed throughout the cytoplasm on EM **(E)**. Paired helical filaments were occasionally seen in pretangle neurons of AD **(F)**. Another pretangle neuron with perinuclear accentuation of tau immunoreactivity (**G**, stacked 3D image; case 2). EM image **(I)** and the corresponding LM counterpart on the same plane **(H)** were compared, QD-decorated straight filaments were seen around the nucleus (N) and some of them were attached to the nuclear membrane at the tip (asterisk, **J**). Scale bars in **A-C, G-I** = 5μm; **D, E, J** = 500 nm; **F** = 100 nm.

Confocal examination of AD-pretangles revealed a mixture of granular tau immunoreactivity and small tangle formation (Figure 3A, stacked 3D image). When an EM section (C) and the corresponding LM counterpart on the same plane (B) were compared, the tangle-like aggregate (B, arrow; C, rectangle d) was found to be a small bundle of straight filaments tightly arranged in parallel (D), which is indistinguishable from mature NFTs in AD (Figure 3B-D). Such a precise comparison on the corresponding planes of LM and EM was not possible between 3D stacked image (A) and the EM section (C). In contrast, granular immunoreactivity on the LM plane (B), corresponded to straight filaments randomly and sparsely distributed throughout the cytoplasm (arrowheads, E), probably representing an earlier stage before bundle formation (Figure 3B, C, E). Occasional paired helical filaments (PHFs) were observed in AD-pretangles (Figure 3F). The distribution of these tau filaments was so sparse that they could be identified on EM only with dual guidance through subcellular orientation using a LM image and QD immunolabeling. Perinuclear staining was sometimes found in AD-pretangles on confocal images (G: stacked 3D image and corresponding plane on LM (H) and EM (I)). This corresponded to a low density of immunolabeled straight filaments attached to the nuclear membrane (Figure 3I, J, asterisk). The diameter of straight filaments ranged from 14 to 16 nm, and the period of filament constriction was 70 to 90 nm in AD and normal aging cases.

### LM findings and corresponding ultrastructures of CBD-pretangles

**Figure 4:**
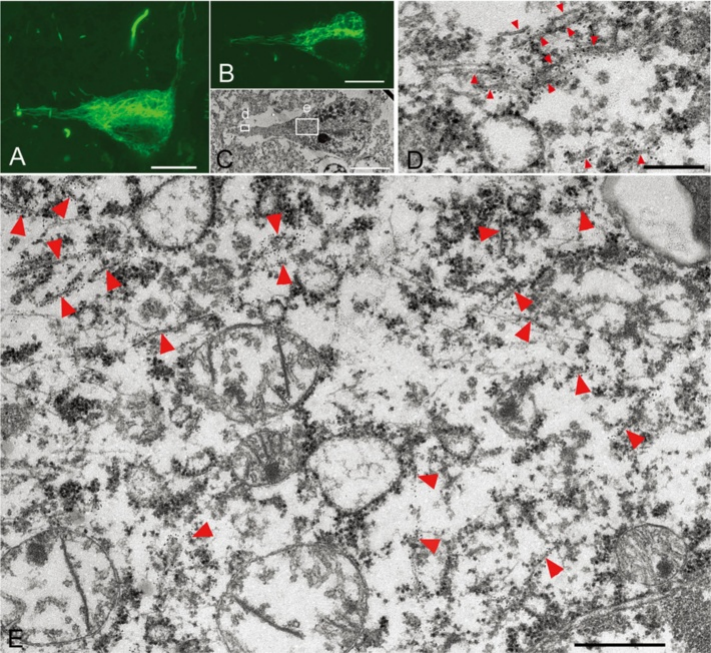
**Correlation of immunolabeled LM and EM images of CBD-pretangles.** Reticular tau immunoreactivity in the cytoplasm in a pretangle neuron of CBD (case 3) labeled with anti-tau antibody (AT8) visualized with QD 655, also labeled with Alexa 488 for more precise confocal images **(A)**. When compared between LM **(B)** and its exact EM counterpart, reticular tau immunoreactivity in the cytoplasm (**C**, rectangle e) was composed of randomly distributed straight filaments (**E**, arrowheads). Tau immunoreactivity in the dendrite (**C**, rectangle d) on LM corresponded to a few 15-nm straight filaments assembled roughly in parallel. Their arrangement was less tight than that in NFTs in AD. Scale bars in **A-C** = 10 μm; **D** = 250 nm; **E** = 500 nm.

**Figure 5:**
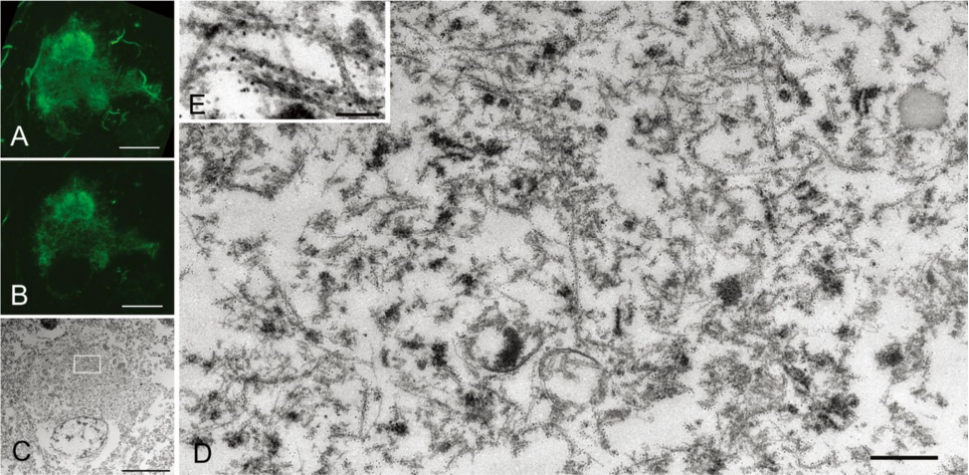
**Correlation of immunolabeled LM and EM images of pretangles in a severe case of CBD.** In a pretangle neuron from a severe case of CBD (case 4), reticular tau immunoreactivity was denser on 3D reconstruction **(A)**. When focusing on the denser area of round inclusion **(B, C)**, the corresponding EM section contained abundant tau-positive straight filaments that did not form a parallel arrangement **(D)**. These straight filaments were thicker with a diameter of 15–20 nm **(E)**. Scale bars in **A-C** = 10 μm; **D** = 500 nm; **E** = 100 nm.

CBD-pretangles, which were often found in the superficial and deep layers of the cerebral cortex, were characterized by diffuse and reticular (rather than granular) immunoreactivity in the neuronal cytoplasm with few solid aggregates (Figure 4A). The density of the reticular structures varied from cell to cell or from case to case. 3D observations using confocal microscopy showed that the reticular structures extended into the distal portions of dendrites (Figure 4A). Perinuclear tau immunoreactivity, as seen in AD-pretangles, was not observed in CBD-pretangles. Correlation of the LM and EM images demonstrated that reticular structures seen by confocal microscopy corresponded to straight filaments, which were diffusely and randomly distributed throughout the cytoplasm and did not displace cellular organellae (Figure 4A-C, E). In dendrites, a few straight filaments were assembled in a roughly parallel fashion. Their arrangement was less tight than in NFTs in AD (Figure 4D). In tissue from the more severe case of CBD (case 4), reticular structures were denser on both confocal microscopy and EM images than in case 3 (Figure 5A- E). Interestingly, straight filaments were thicker in diameter in case 4 (15–20 nm) than in case 3 (14 to 15 nm). Straight filaments also appeared longer in case 4. In this study, PHFs were not observed in CBD-pretangles in either case.

### Ultrastructure of Pick-like inclusions in small neurons in two CBD cases

**Figure 6:**
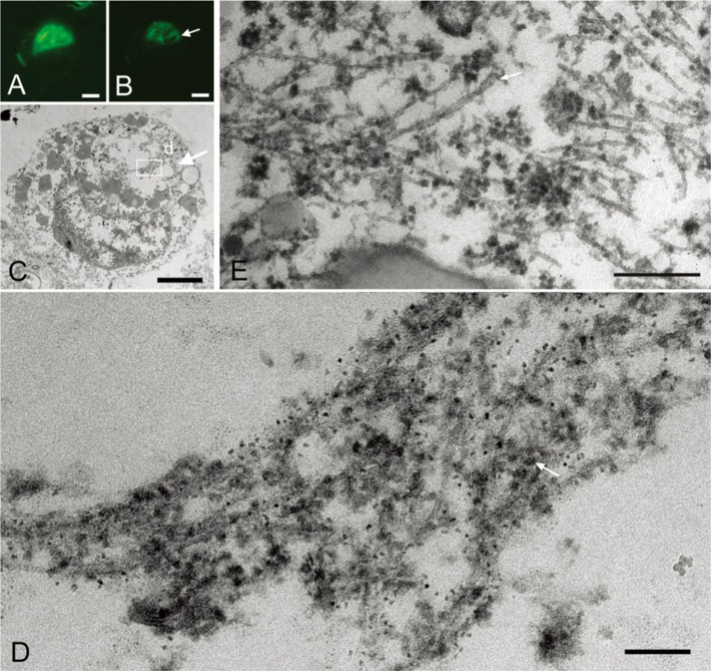
**Correlation of immunolabeled LM and EM images of Pick-like inclusions in two CBD cases.** A densely packed round inclusion (Pick-like inclusion) from a CBD case (case 3) labeled with anti-PHF antibody (AT8) visualized with QD 655, also labeled with Alexa 488 for more precise confocal images **(A)**. Correlated LM and EM images **(B, C)** showed that tau immunoreactivity around the cavity on LM corresponded to bundles of tau filaments that were not arranged as parallely as in AD (**C**, rectangle d; **D**). Note that these filaments were intermingled with ribosomes (**D**, arrow). **(E)** The ultrastructure of Pick-like inclusions in another case of CBD (case 4) also revealed randomly assembled tau filaments with occasional formation of paired helical filaments (a periodicity of 130 nm, arrow). Scale bars in **A** to **C** = 3 μm; **D** = 50 nm; **E** = 100 nm. **A** to **D**, case 3; **E**, case 4.

Densely packed round inclusions (Pick-like inclusions) were found mainly in the small neurons in the superficial layer of the cerebral cortex of CBD cases; their tau immunoreactivity was more compact and denser than that of CBD-pretangles. On confocal observation, we found that these inclusions often contained small cavities (Figure 6A-B). The correlation of LM and EM images revealed bundles of tau filaments around the cavities (Figure 6B-D). Tau-positive filaments were composed of straight filaments and PHFs with a periodicity of approximately 130 nm (Figure 6D-E). These filaments were loosely assembled and usually intermingled with cellular organellae, especially ribosomes (Figure 6D, arrow). Strictly speaking, the straight filaments were not oriented parallel to each other. The diameters of straight filaments in these inclusions ranged from 13 to 15 nm in case 3 and 15 to 16 nm in case 4. Similarities and differences among AD-pretangles, NFTs in AD, CBD-pretangles, and Pick-like inclusions in CBD are listed in Table 2.

**Table 2:** **Similarities and differences between AD-pretangle and CBD-pretangle**

		**AD**		**CBD**	
		**Pretanle**	**NFT**	**Pretangle**	**Pick like inclusion**
LM findings (confocal images)	Morphology	Granular	Fibrillary	Reticular	Round, frequent vacuoles
	Perinuclear accentuation	Occiasional	Occasional	None	None
	Size of neurons involved	Small- to large- sized	Small- to large- sized	Medium- to large-sized	Small-sized
EM findings	Density of tau filaments	Very sparse*1	Very dense*2	Sparse	Dense
	Arrangement of tau filaments	Irregular/regular (focal NFT formation*3)	Regular (NFT formation)	Irregular	Irregular
	Diameter of straight filaments	About 15 nm	About 15 nm	14-20 nm	About 15 nm
	PHF (a periodicity)	Occasional (about 80 nm)	Frequent (about 80 nm)	None	Occasional (130–180 nm)

IHC, immunohistochemistry; LM, light microscopy; EM, electron microscopy; PHF, paired helical filaments; *1, Density of straight tau filament is more sparse in AD-pretangles than in CBD-pretangles; *2, Density of tau filaments is more dense in AD-NFT than in Pick-like inclusions of CBD; *3, NFT, neurofibrillary tangle signifying a regularly and tightly arranged bundle of straight or paired helical filaments.

Among hundreds of AT8-positive neurons on confocal, 3–4 pretangles were selected in each AD and CBD case, which were 3D-reconstructed and prepared for EM observation. In addition, 3–4 Pick-like inclusions were selected in each CBD case and were processed similarly.

## Discussion

The name **‘**pretangle’ was originally used to describe the premature stage of NFT formation in AD [2-4]. However, in CBD, similar structures (also called ‘pretangles’) are more prevalent than NFTs in the cerebral cortex [6]. It has been unclear whether the pretangles of CBD represent a premature stage before NFT formation and whether they are different from AD-pretangles. Because pretangles are defined only by LM findings [2-4], it would be helpful to compare the corresponding pretangle ultrastructures between the two diseases. However, CBD pretangles in the cortex have not previously been described at the EM level [22-28]. To address this issue, we used a method of correlative light and electron microscopy with QD immunolabeling [19]. This procedure allowed us to observe not only the features of filamentous structures of inclusions but also their intracellular distribution and relationship with cellular organellae.

Using correlated LM and EM images, we observed a distinctive EM feature of AD-pretangles: specifically, a strong tendency to form bundles as a precursor to NFTs. Even in the earliest stages of tau accumulation, small pieces of NFTs could already been seen on the background of diffuse and granular tau staining on LM (Figure 3A) [3,29]. Correlation of the LM and EM images revealed that the granular cytoplasmic staining on LM represented straight filaments (sparsely distributed in the neuronal cytoplasm), and the small tangles represented small bundles of parallel filaments (Figure 3D). Very similar EM findings were reported by Bancher [2], although it remains unclear whether their EM findings really represented LM-defined pretangles or not. Because our method of 3D-oriented immunoEM not only identify pretangles on LM, it is quite sure that our immuno EM findings represent LM-identified pretangles. Moreover, it is further possible to correlates the EM plane (Figure 3C) and its exact counterpart of the corresponding LM plane (Figure 3B), even a small aggregates (Figure 3B arrow) not identifiable on 3D stack image (Figure 3A) can be examined with EM (Figure 3C) for comparison with LM at the extreme accuracy. Another feature of AD-pretangles was perinuclear accumulation of tau. Correlation with its EM counterpart showed that a small number of tau-positive 15-nm straight filaments were present around the nuclear membranes of AD-pretangles (Figure 3G-J). It has been reported that PHF or tau-like immunoreactivity may be present in close proximity to the nuclear membrane of mature NFTs in AD [29-32], and this report is the first demonstration of tau-immunolabeled filaments around the nuclear membrane of AD-pretangles. Although intranuclear processes such as aberrant cell cycling may be related to the pathogenesis of AD [33,34], it is unclear how these processes are related to the perinuclear or cytoplasmic deposition of tau.

A similar approach to CBD-pretangles of the cerebral cortex revealed several findings that differed from the features of AD-pretangles and NFTs: (i) random and diffuse distribution of 14–20 nm straight filaments and (ii) paucity of PHFs and fibrillary bundle formation. These ultrastructural architectures may explain the reticular or diffuse tau immmunoreactivity of CBD-pretangles seen on LM (Figures 4 and 5). In dendrites, a small number of straight filaments were observed lying parallel to the dendritic shaft (Figure 4D), similar to the previous reports of dendritic lesions in CBD [8,22]. Even in CBD-pretangles with abundant tau filaments, this random and diffuse distribution of straight filaments was maintained with little NFT formation (Figure 5D). Indeed, this architecture was maintained even in Pick-like inclusions, where tau filaments were randomly assembled and were composed mainly of straight filaments and, to a lesser extent, PHFs with a periodicity of 130–180 nm (Figure 6D-E). Thus, so-called CBD-pretangles are a random accumulation of tau-positive straight filaments, rarely evolving into so-called NFTs even when the filament density is increased. These findings, especially regarding the filamentous structures themselves, were similar to previous findings in CBD (15–20 nm straight filaments or PHFs with a periodicity of 120–180 nm), which were observed in Pick-like inclusions [22-24,27], ballooned neurons [24,28], neuronal inclusions in the brainstem [22,26,28], or an *in vivo* study using CBD brains [25]. However, this study is the first to clarify the EM structures of cortical pretangles in CBD by accurately correlating them with LM images. Authentic Pick bodies in Pick body disease were more solid than pretangles on LM, where abundant tau-positive fibrils, 15 nm in diameter, were randomly arranged without forming PHF [19].

In this study, we greatly enhanced the reliability of pre-embedding immunoEM using QDs. Although QDs are considered suitable for CLEM, their reliability as a reporter for ultrastructural immunolabeling has been debated. The penetration of QD labeling is reported to be limited to several micronmeters from the sample surface [15]. However, we were successful in immunolabeling 25-μm-thick free-floating sections by increasing the incubation time with QD-conjugated secondary antibodies to 8 hr at room temperature. This procedure enabled us to label the entire thickness of floating sections with QDs so that each tau filament was sufficiently labeled (Figures 3 and 4). Consequently, confocal images and immunoEM images could be tightly correlated.

Other disadvantages of QDs are that they have a lower electron density and less distinct contours than colloidal gold for immunolabeling. We previously used EDX spot analysis with STEM to demonstrate the presence of Se and Cd on a pixel basis [19]. This EDX spot analysis, now extended to map the entire EM field, resulted in clear visualization of the position and form of each QD particle. When the corresponding EM image was overlaid, QDs could be readily differentiated from the grayscale cellular backgrounds (e.g., ribosomes) (Figure 2). Similar elemental mapping of Cd has been reported using electron energy loss spectrography (EELS) to detect QDs in ultrathin EM samples [35]. However, compared with EELS, EDX is more suitable for the detection of heavy metals, such as Cd or Se [19,36]. Moreover, because EELS is performed without electron staining, it is difficult to gain sufficient contrast in EM images [35]. Therefore, combined with pre-embedding Q-dot immunoEM and EDX mapping, the use of QDs is one of the most sensitive and distinct ultrastructural immunolabeling techniques available and might be particularly suitable for the correlation of LM/EM images.

## Conclusions

Accurate identification of pretangles on LM, followed by EM examination of their exact counterpart was achieved through tau immunolabeling with QD, fluorescent nanocrystals, which are detectable with LM (fluorescence signal) and with EM (electron dense particles with halo). EDX spot analysis to confirm the identity of QD on EM section by showing energy peaks for Cd and Se is now extended to map the entire EM field to highlight QD particles. This improved method with EDX mapping clearly demonstrated for the first time that AD-pretangles showed a strong tendency to form fibrillary tangles even at an early stage, whereas pretangles or Pick-like inclusions in tissue from patients with CBD did not even at an advanced stage. This novel strategy is useful to clarify how molecules other than tau are organized into ultrastructures in the early stages of disease-specific lesions.

## Additional files

Additional file 1: Figure S1. Optimal of dilution of QD-conjugated secondary antibodies for ultrastruc-tural immunolabeling. Additional file 2: Figure S2. Energry dispersive X-ray (EDX) mapping of Quantum dots (QDs).
