# Supplementary material for: Ultrastructural differences in pretangles between Alzheimer disease and corticobasal degeneration revealed by comparative light and electron microscopy
Source: Acta Neuropathol Commun. 2014 Dec 11;2:161. doi: 10.1186/s40478-014-0161-3 (PMC4269873; doi:10.1186/s40478-014-0161-3)
Supplement: Additional file 1: Figure S1. — Optimal of dilution of QD-conjugated secondary antibodies for ultrastruc-tural immunolabeling. [file 40478_2014_161_MOESM1_ESM.jpeg]

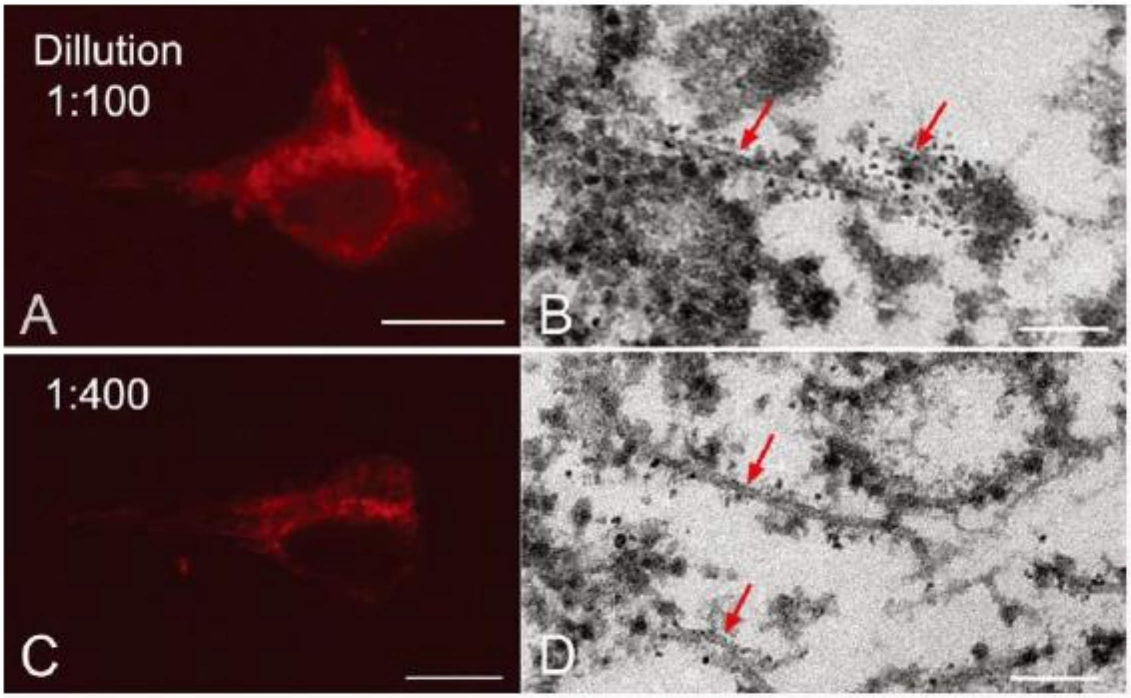

Additional file 1: Figure S1 Optimal of dilution of QD-conjugated secondary antibodies for ultrastructural immunolabeling.

Immunolabeling with an anti-PHF tau (AT8, 1:700) is visualized with Quantum dot (QD) 655-conjugated secondary antibody at a dilution of 1:100 (A, B) or 1:400 (C, D). At 1:100, its fluorescence signal (A) was intense, while QD labeling on fibrils (B, arrows) was so excessive that may mask underlying ultrastructure. At 1:400, its QD labeling on fibrils (D: arrows) was adequate, while its fluorescent signal was weak. Therefore, colabeling with Alexa488 was performed to ensure intense fluorescent signal to reconstructe3D data set. CBD (case 3). Scale bars = 10 μm (A, C), 10 nm (B, D) .
