# Supplementary material for: Ultrastructural differences in pretangles between Alzheimer disease and corticobasal degeneration revealed by comparative light and electron microscopy
Source: Acta Neuropathol Commun. 2014 Dec 11;2:161. doi: 10.1186/s40478-014-0161-3 (PMC4269873; doi:10.1186/s40478-014-0161-3)
Supplement: Additional file 2: Figure S2. — Energry dispersive X-ray (EDX) mapping of Quantum dots (QDs). [file 40478_2014_161_MOESM2_ESM.jpeg]

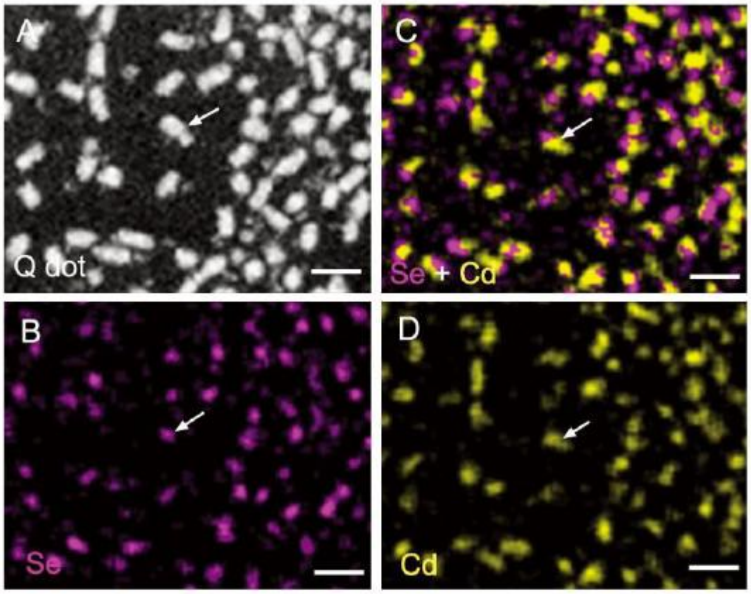

Additional file 2: Figure S2 Energry dispersive X-ray (EDX) mapping of Quantum dots (QDs). Ultrastructure of QDs, dribbled on the formvar membrane, are electron-dense and oblong with homogeneous diameter (A). Pixel-based EDX elementary mapping highlighted the distribution of Se (pink, B) or Cd (yellow, D), which exactly correspond to the ultrastructural shape of QDs (C, an arrow). Scale bars = 20 nm.
